# Nanomedicine in Pancreatic Cancer: Current Status and Future Opportunities for Overcoming Therapy Resistance

**DOI:** 10.3390/cancers13246175

**Published:** 2021-12-07

**Authors:** Michelle K. Greene, Michael C. Johnston, Christopher J. Scott

**Affiliations:** 1 The Patrick G Johnston Centre for Cancer Research, School of Medicine, Dentistry and Biomedical Sciences, Queen’s University Belfast, Belfast BT9 7AE, UK; michael.johnston@qub.ac.uk (M.C.J.); c.scott@qub.ac.uk (C.J.S.); 2 Elasmogen Ltd., Liberty Building, Foresterhill Health Campus, Foresterhill Road, Aberdeen AB25 2ZP, UK

**Keywords:** nanomedicine, pancreatic cancer, resistance

## Abstract

**Simple Summary:**

Despite access to a vast arsenal of anticancer agents, many fail to realise their full therapeutic potential in clinical practice. One key determinant of this is the evolution of multifaceted resistance mechanisms within the tumour that may either pre-exist or develop during the course of therapy. This is particularly evident in pancreatic cancer, where limited responses to treatment underlie dismal survival rates, highlighting the urgent need for new therapeutic approaches. Here, we discuss the major features of pancreatic tumours that contribute to therapy resistance, and how they may be alleviated through exploitation of the mounting and exciting promise of nanomedicines; a unique collection of nanoscale platforms with tunable and multifunctional capabilities that have already elicited a widespread impact on cancer management.

**Abstract:**

The development of drug resistance remains one of the greatest clinical oncology challenges that can radically dampen the prospect of achieving complete and durable tumour control. Efforts to mitigate drug resistance are therefore of utmost importance, and nanotechnology is rapidly emerging for its potential to overcome such issues. Studies have showcased the ability of nanomedicines to bypass drug efflux pumps, counteract immune suppression, serve as radioenhancers, correct metabolic disturbances and elicit numerous other effects that collectively alleviate various mechanisms of tumour resistance. Much of this progress can be attributed to the remarkable benefits that nanoparticles offer as drug delivery vehicles, such as improvements in pharmacokinetics, protection against degradation and spatiotemporally controlled release kinetics. These attributes provide scope for precision targeting of drugs to tumours that can enhance sensitivity to treatment and have formed the basis for the successful clinical translation of multiple nanoformulations to date. In this review, we focus on the longstanding reputation of pancreatic cancer as one of the most difficult-to-treat malignancies where resistance plays a dominant role in therapy failure. We outline the mechanisms that contribute to the treatment-refractory nature of these tumours, and how they may be effectively addressed by harnessing the unique capabilities of nanomedicines. Moreover, we include a brief perspective on the likely future direction of nanotechnology in pancreatic cancer, discussing how efforts to develop multidrug formulations will guide the field further towards a therapeutic solution for these highly intractable tumours.

## 1. Introduction

Pancreatic cancer (PaCa) is a highly aggressive malignancy that is almost universally fatal. Of the various types of pancreatic tumours, the majority are classified as ductal adenocarcinomas that originate in the enzyme-producing exocrine tissues [1,2]. The prognosis for these tumours is exceptionally poor, with a 5-year survival rate of approximately 5% that has remained virtually unchanged over several decades [3]. This dismal outlook is partly attributed to a largely asymptomatic presentation and a lack of detection biomarkers, meaning that diagnoses are often not confirmed until an advanced stage when curative surgical resection is no longer feasible. Treatment options are then mainly confined to conventional chemotherapy that typically involves a combination of two or more agents. However, even if patients can endure the debilitating side-effects of these drug regimens, they are only likely to experience marginal and transient improvements at best as resistance mechanisms often prevail. Thus, there is an urgent unmet need for more effective strategies to treat PaCa.

**Figure 1:**
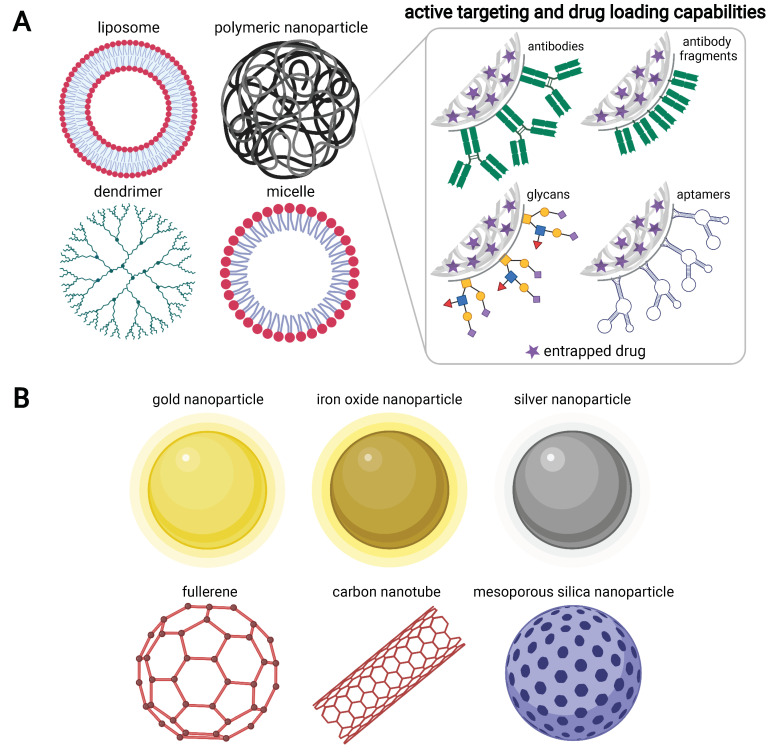
Selected examples of organic and inorganic nanoparticles. (**A**) Organic materials used for nanoparticle construction include lipids and polymers of natural or synthetic origin. Nanoparticles within this class are commonly exploited as drug delivery vehicles and may be functionalised with ligands to enhance targeting to cell surface receptors, as shown for polymeric nanoparticles. (**B**) Inorganic materials used for nanoparticle construction include carbon, silica and metals such as gold, silver and iron oxide. Nanoparticles within this class are commonly exploited for diagnostic and imaging purposes, although they may also be deployed as therapeutics given their capacity for drug loading and their unique physicochemical properties that facilitate approaches such as photothermal tumour ablation. As illustrated in (**A**), targeting ligands may be similarly conjugated to inorganic nanoparticles. Although selected examples are shown, many other types of organic (e.g., polymersomes and solid lipid nanoparticles) and inorganic (e.g., quantum dots and lanthanide-doped upconversion nanoparticles) platforms have been developed for wide-ranging biomedical applications. Much diversity exists between the various types of nanoparticles, particularly with regard to size. For example, diameters of <100 nm are typically observed for dendrimers, micelles and gold nanoparticles, whereas liposomes and polymeric nanoparticles often measure >100 nm. Many of the clinically approved nanomedicines fall within the latter size range, with diameters of 110 nm and 130 nm reported for Onivyde and Abraxane, respectively.

In the strive to address this need, nanomedicine is rapidly gaining traction as a leading approach with much potential to reform the management of PaCa [4,5,6,7,8,9,10,11,12,13,14,15,16]. This discipline involves the medical application of nanoparticle technologies, which have undergone an extensive and vibrant history of development spanning many disease settings. Most recently, this has included the ongoing SARS-CoV-2 pandemic, where nanoparticles have earned widespread recognition for their integral contribution to vaccine design [17,18]. Although a consensus definition for nanoparticles has not been established, they are generally considered as having at least one dimension in the range of 1–100 nm and encompass a broad range of platforms with diverse structures, material composition and physicochemical properties (Figure 1). 

Efforts to develop nanoparticles have largely concentrated on oncology indications, where they have demonstrated much promise in tumour imaging, diagnosis and treatment [19,20,21]. In particular, their ability to bypass mechanisms of tumour resistance to therapy has been a key driving factor in their success [22,23,24,25,26]. Several nanoformulations have progressed to clinical evaluation in PaCa patients, of which two have been approved to date. Firstly, Abraxane, which is an albumin-bound nanoformulation of paclitaxel, gained FDA authorisation in 2013 as a first-line treatment for metastatic PaCa in combination with gemcitabine. The basis for this approval was founded on the results of the phase 3 MPACT trial, which showed an enhancement in overall survival upon addition of Abraxane to standard-of-care gemcitabine monotherapy [27]. Secondly, and more recently, positive outcomes from the phase 3 NAPOLI-1 trial highlighted the clinical efficacy of a liposomal preparation of irinotecan in PaCa patients, marketed under the trade name Onivyde [28]. Based on these observations, the FDA sanctioned the use of Onivyde in 2015 as a second-line option alongside 5-fluorouracil (5-FU) and leucovorin in metastatic PaCa patients that have responded poorly to gemcitabine alone. Despite the seemingly modest impact on survival in both trials (8.5 vs. 6.7 months in MPACT and 6.1 vs. 4.2 months in NAPOLI-1), these improvements were in fact highly encouraging given that the patient cohorts had already experienced metastatic onset, at which stage the median duration of survival can be as little as two months [29]. The approval of Abraxane and Onivyde has now propelled nanomedicine to the forefront of clinical management strategies for PaCa, providing patients with a wider repertoire of treatment options and stimulating intense efforts to further build upon this success. Below, we provide an overview of the various benefits afforded by nanomedicine that have attracted widespread interest throughout the scientific community. We discuss attempts to exploit nanotechnology for PaCa therapy, focusing on those studies where the unique features of nanoparticles have been leveraged to override drug resistance.

## 2. Advantages of Nanoparticle-Based Therapies

Nanoparticles possess a number of desirable attributes that have led to their extensive application throughout the field of oncology. One of the primary motives for the use of nanoparticles in cancer therapy stems from their exceptional utility as drug delivery vehicles [30,31]. Many of the constraints of conventional drug administration can be overcome through entrapment within nanoparticles, such as poor aqueous solubility [32], unfavourable pharmacokinetics [33,34,35,36,37], unacceptable toxicity [38,39], and limited stability due to metabolic or enzymatic degradation [40,41,42]. Moreover, with cancer therapy typically involving combinations of drugs with dissimilar pharmacology, this can complicate dosing and hinder efforts to coordinate their tumour delivery at synergistic ratios. These issues can be alleviated through co-formulation within a single nanoparticle platform to synchronise the pharmacokinetics and biodistribution of each component drug [43,44]. 

Drug delivery to tumours can also be enhanced using nanoparticles [45,46], which demonstrate a unique ability to exploit the ‘leaky’ vasculature and impaired lymphatics that are often found in undifferentiated tumour tissues. These characteristics facilitate the ‘passive’ accumulation of nanoparticles within tumours, allowing payloads to be preferentially deposited at the target site in a phenomenon known as the ‘enhanced permeability and retention (EPR) effect’ [47,48,49]. Tumour retention of nanoparticles can then be further improved through surface functionalisation with targeting ligands such as antibodies and peptides [50,51]. This concept, referred to as ‘active’ targeting, promotes nanoparticle engagement with tumour cells and has been widely applied to many receptor–ligand pairings [52,53,54,55,56,57,58,59]. In addition, it is now possible to engineer ‘smart’ nanoparticles that release their payload upon stimulation, providing yet another level of control over tumour drug delivery. Common stimuli employed to date include the external application of light [60,61,62,63], ultrasound [64,65] or X-ray irradiation [66]. Alternatively, drug release may be triggered endogenously through construction of nanoparticles with pH- [67], redox- [68,69,70], hypoxia- [71,72] or enzyme-responsive [73] properties. The combined potential for tumour-selective drug delivery via these mechanisms can lead to several benefits, including a reduction in off-target effects that often manifest as intolerable, treatment-limiting toxicities in those receiving conventional therapy [74,75,76]. 

Other benefits can be derived from the diverse materials used to fabricate nanoparticles. Phospholipids show excellent biocompatibility and biodegradability and can be readily assembled into liposomal vesicles with high drug-loading capacity, which constitute the bulk of clinically approved nanomedicines to date. While metallic nanoparticles can provide useful contrast enhancement for imaging and diagnostic purposes, they are also highly valued as therapeutics due to their unique thermal [77,78,79,80,81] and radiosensitising [82] capabilities [83]. With the use of polymers for nanoparticle construction, controlled release of entrapped cargo is possible and can be tailored to the required specification. For example, polylactic-co-glycolic acid (PLGA) contains biodegradable ester groups and its degradation rate is largely dictated by the ratio of its component monomers. For PLGA composed of a 50:50 monomeric ratio, hydrolytic breakdown will occur more rapidly compared to other grades containing a higher proportion of either of the two units [84]. Thus, the release kinetics of nanoencapsulated drugs may be finely tuned through alteration of the type or composition of polymer. Moreover, hydrophilic polymers can impart a ‘stealth’ coating onto nanoparticles that effectively conceals them from immune recognition as foreign material. Polyethylene glycol (PEG) is particularly effective in this context and is routinely incorporated into most present-day nanoformulations [85]. Taken together, it is clear that these materials, alongside many others, each offer unique properties that allow for significant versatility in nanoparticle design and application.

## 3. Addressing Therapy Resistance in PaCa Using Nanoparticle-Based Platforms 

Pancreatic tumours are renowned for their intractable response to many therapeutic interventions, including molecularly targeted agents, chemotherapy and radiotherapy. The processes that contribute to the treatment-refractory nature of PaCa are multifactorial, involving both intrinsic features of the tumour cell population, together with extrinsic factors from the surrounding microenvironment [86,87,88]. These can either pre-exist prior to therapy induction, or instead emerge after an initial period of treatment sensitivity, representing *primary* or *acquired* mechanisms of resistance, respectively. In the following sections, we outline several resistance traits of pancreatic tumours that predispose patients to therapy failure and discuss how these can be effectively surmounted through use of nanotechnology. While radioresistance is a common feature of PaCa and there are nano-based strategies to tackle this issue, it is beyond the scope of this current article and discussed in more detail elsewhere [89,90,91]. 

### 3.1. Alterations in Drug Transport

Insufficient drug uptake and/or enhanced drug efflux represent two of the most common chemoresistance mechanisms in PaCa that can particularly impact upon frontline therapy with gemcitabine. As a hydrophilic molecule, gemcitabine does not readily diffuse across the lipid bilayer of cell membranes and is thus dependent on the presence of nucleoside transporters for intracellular entry. These include members of the solute carrier (SLC) superfamily, such as the human concentrative nucleoside transporters (hCNT) and the human equilibrative nucleoside transporters (hENT), with reports suggesting that hENT1 is primarily responsible for gemcitabine uptake [92,93,94]. However, downregulation of nucleoside transporters such as hENT1 is frequently observed in PaCa and has also been shown to correlate with reduced survival in gemcitabine-treated patients [95,96,97]. This resistance mechanism may be effectively bypassed through drug formulation within nanoparticles, whose internalisation is not contingent on nucleoside transporter expression but instead proceeds via pathways such as macropinocytosis and clathrin-mediated endocytosis, amongst others [98,99]. In agreement, Guo and co-authors showed that hENT1 inhibition in PaCa cells reduced their sensitivity to gemcitabine, which could be restored through nanoformulation of the drug [100]. In further studies, nanoencapsulated gemcitabine has also demonstrated superior tumour cytotoxicity over free drug, again indicating that drug intracellular uptake can be enhanced through packaging within nanoparticles [101,102,103].

As with uptake transporters, alterations in the expression and activity of transmembrane efflux pumps can contribute to chemoresistance. Cellular drug expulsion is predominantly mediated by the adenosine triphosphate-binding cassette (ABC) superfamily, encompassing members such as P-glycoprotein, multidrug resistance proteins and breast cancer resistance protein [104]. These proteins counteract the inward diffusion of molecules across the lipid bilayer by shuttling them back into the extracellular space, leading to a reduction in the intracellular drug reservoir that limits therapeutic efficacy. On the contrary, nanoparticle-based drug vehicles tend not to be recognised by efflux pumps and so provide a highly effective means of subverting this resistance mechanism. In support of this, photodynamic treatment efficacy was shown to negatively correlate with ABCG2 expression in PaCa models, which could be overcome by entrapping the photosensitiser within polymeric nanoparticles [105].

Nanoparticles have also been exploited as carriers for therapeutics that can modulate the expression and activity of membrane transporters [106,107]. Many of these studies have involved the delivery of nucleic acids such as small interfering RNA (siRNA) and antisense oligonucleotides, which are ideal candidates for nanoencapsulation since this can protect against premature degradation by nucleases in the circulation [108]. In addition, nanotechnology can overcome many limitations of viral-based vectors that have traditionally been used for delivery of genetic material, such as low packaging capacity and immunogenicity. Several reports have demonstrated efficient knockdown of efflux transporter expression in tumour cells using nano-enabled approaches, resulting in enhanced sensitivity to co-encapsulated chemotherapies [109,110,111]. Alternatively, similar principles could be applied to reconstitute the expression of uptake transporters in PaCa cells; for example, through encapsulation of a hENT1-encoding DNA plasmid. Thus, formulation of dual-loaded nanoparticles, combining standard-of-care agents such as gemcitabine with chemosensitisers that regulate bidirectional drug flux across the cell membrane, represents a promising avenue for future exploration in the PaCa setting.

### 3.2. Hypoimmunogenicity

Unlike other ‘hot’ tumour types such as melanoma and lung carcinoma, PaCa is renowned for its ‘cold’ immune contexture that markedly impairs response to revolutionary immuno-oncology (IO) therapies such as checkpoint inhibitors. This characteristic largely results from the low mutational burden and limited neoantigen expression of pancreatic tumours and is further exacerbated by the immunosuppressive actions of resident stromal cells, as discussed in subsequent sections. Efforts to stimulate tumour immunity include the application of chemotherapeutics that are known inducers of immunogenic cell death (ICD) [112]. For example, polymeric nanoformulations of oxaliplatin and doxorubicin were shown to upregulate the expression of damage-associated molecular patterns (DAMPs) in PaCa models, which triggered dendritic cell maturation and downstream engagement of adaptive immune elements [103]. Both nanoformulations repressed the growth of established Pan02 allografts and were also successfully deployed as part of a prophylactic vaccine strategy. Here, prior inoculation of animals with nanoparticle-treated PaCa cells offered superior protection against a subsequent tumour re-challenge, when compared to free drug treatment. Clearly, vaccines represent a foremost solution to overcome the hypoimmunogenicity of PaCa and it is evident from these studies and others [113,114] that nanotechnology can significantly bolster their immunostimulatory effects. Beyond this unique ability, nanoparticles can also protect labile vaccine components such as peptides and nucleic acids from degradation [115], can be formulated using materials with adjuvant properties [116], and can efficiently target antigen-presenting cells through active or passive means [117]. Despite these advantages, vaccine-based approaches for PaCa have shown limited integration of nanotechnology to date. However, the widespread promise shown by nanovaccines in other tumour settings [118,119,120,121] provides much incentive for future translation to PaCa models. 

### 3.3. Stromal Desmoplasia

**Figure 2:**
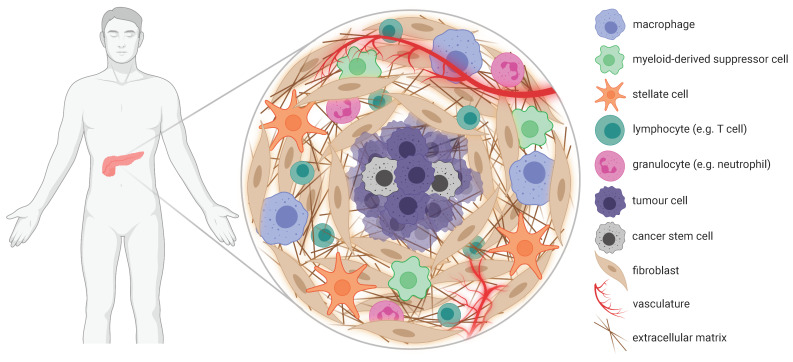
Cellular and acellular elements of PaCa stroma with established roles in treatment resistance. PaCa is characterised by an abundant desmoplastic stroma populated by non-malignant cells such as macrophages, fibroblasts and regulatory T cells. The biological actions and interplay of these cells create a nurturing environment for tumour growth that can markedly limit the effectiveness of therapy. Response to treatment is further impaired by a lack of functional vasculature and fibrotic deposits of ECM throughout the stroma, which pose physical barriers to drug delivery. Collectively, these features highlight the significance of the stroma as a rich source of actionable targets for overcoming PaCa resistance.

A defining feature of PaCa is the development of an extensive fibrotic stroma, known as desmoplasia, which can account for up to 90% of the total tumour mass [122] (Figure 2). This microenvironment is composed of multiple non-neoplastic cell subsets embedded within largely impenetrable networks of extracellular matrix (ECM) proteins, such as collagen, fibronectin and hyaluronan. Collectively, these elements confer resistance to treatment via multiple physical and biological mechanisms, which constitute targets for nanomedicine-based therapeutic strategies, as discussed below. 

#### 3.3.1. Physical Barriers

Pancreatic tumours are often poorly perfused due to their inherent hypovascularity, which forms a major impediment to drug delivery. This challenge is exacerbated by dense deposits of ECM and unchecked growth of tumour cells in a compact tissue space, creating overwhelming stresses and compressive forces that render much of the vasculature non-functional due to collapse. Moreover, plasma leakage from hyperpermeable vessels is not effectively drained by the dysfunctional tumour lymphatics, leading to further increases in interstitial pressure. Together, these abnormalities contribute to treatment resistance by hindering multiple aspects of drug transport, such as delivery into the tumour, extravasation across the endothelium and passage through the interstitium [123].

Attempts to counteract this pathophysiology include strategies aimed at disrupting the stroma to provide a window of opportunity for enhanced drug penetrance [124,125,126,127,128,129,130]. Fibroblasts, and their pancreatic stellate cell (PSC) precursors, have formed common targets for such approaches, given their instrumental role in orchestrating the desmoplastic response through production of ECM elements. These cells exist in a quiescent state under homeostatic conditions; becoming activated during pancreatic carcinogenesis in response to paracrine and contact-mediated cues from tumour cells and other stromal constituents. Nano-inspired efforts to interfere with PSC and fibroblast activation have demonstrated success through multifaceted mechanisms such as PI3K or MAPK inhibition [131,132], lipid regulation [133], Wnt 16 downregulation [134] and reconstitution of relaxin or TRAIL expression [135,136]. However, most studies have focused on the TGF-β and Shh signalling cascades, which play a major role in shaping the pro-fibrotic secretome of these cells. Nanoformulated inhibitors targeting both pathways have been shown to downregulate PSC and fibroblast activation markers in PaCa models, leading to remodelling of the stromal architecture that enhances the delivery and efficacy of chemotherapeutics [137,138,139,140,141,142,143]. For example, stromal collagen content was significantly reduced by polymeric micelles encapsulating the Shh inhibitor vismodegib, resulting in higher intratumoral deposition of a co-entrapped SN-38 payload and greater retardation of tumour growth [144]. With data also confirming controlled release of SN-38 from these micelles, this further highlights the benefits of nanomedicine in ensuring sustained exposure of tumour cells to therapeutic drug levels that discourages the selection of resistant clones. 

Despite these promising preclinical observations, stromal depletion has yielded disappointing outcomes in PaCa patients, and has now become highly contentious following conflicting evidence that it can enhance tumour aggressiveness and metastatic dissemination [145,146,147,148,149,150]. These insights emphasise the need for caution when disrupting the stroma, which is now being reflected in the latest nanomedicine research. For example, Chen et al. exploited the pH differential throughout the stroma to selectively disrupt the inner core through triggered release of nanoencapsulated drugs under acidic conditions, whilst simultaneously preserving the outermost regions [151]. This work provides an excellent example of how unique stimuli-responsive properties may be integrated into nanoparticles to allow careful disruption of defined areas of the stroma, ensuring that any protective role of the stroma in restraining PaCa is not compromised.

Other efforts to overcome the physical barriers posed by the PaCa microenvironment have focused on targeting the vasculature to restore vessel patency and intratumoral drug perfusion [152,153]. Although the hyperpermeability of neovasculature can favour drug access to solid tumours, the endothelial lining of PaCa vessels often features a relatively high coverage of pericytes. These cells can block vascular fenestrations and thus impede the extravasation of drugs from the circulation into the tumour bed. With research indicating that TGF-β signalling plays a dominant role in mediating pericyte interactions with endothelial cells [154], interference with this pathway could potentially enhance tumour drug entry. This strategy was pursued by Meng et al., who showed that complexation of a TGF-β inhibitor to mesoporous silica nanoparticles could perturb the colocalisation of pericytes and endothelial cells [155]. Pre-treatment of pancreatic BxPC-3 xenografts with these nanoparticles led to enhanced penetrance of a sequentially administered nanoformulation of gemcitabine, resulting in significant retardation of tumour growth. Notably, free versus nanoformulated drug comparisons confirmed the superior ability of the latter to overcome this chemoresistant property, with the added benefit of reduced toxicity.

#### 3.3.2. Biological Barriers Leading to Immunosuppression

Even if physical hurdles can be overcome, therapeutics often encounter further resistance arising from the complex and dynamic biological interplay between stromal elements and the neoplastic epithelium. Key players in this respect are the cell subsets that populate the stroma, comprising fibroblasts as discussed previously, as well as immune cells from both lymphoid and myeloid lineages for example. Much progress has been made in unravelling the molecular cross-talk and communication channels that exist between these cells, which has highlighted their pivotal involvement in tumour progression and resistance to therapy [156]. In particular, accumulating evidence points to their influential role in sculpting the immunosuppressive microenvironment of PaCa that can desensitise patients to IO therapies. This knowledge has informed the design of therapeutic strategies focused on modulating stromal cell biology [157,158], many of which have been facilitated by nanotechnology.

In addition to their role in driving desmoplasia, fibroblasts can contribute to therapy resistance by dampening immune surveillance in the pancreatic tumour microenvironment. One of the key mechanisms by which they achieve this is via secretion of the CXCL12 chemokine, which facilitates immune evasion by blocking T cell trafficking to the vicinity of tumour cells [159]. To intervene with this process, nanoparticles have been designed to restore antitumour immunity through sequestration of CXCL12 [160,161]. These comprise of liposome-encapsulated plasmid DNA encoding a fusion protein that binds to CXCL12 with high affinity. Treatment with this formulation trapped CXCL12 and altered the immune landscape of orthotopic KPC allografts, most notably through the induction of a pronounced CD8+ T cell infiltrate that primed tumours for checkpoint inhibition with a co-delivered PDL1 trap. In contrast, combination trap therapy in a non-encapsulated format showed limited efficacy, confirming an important role for nanotechnology in overcoming the intractability of PaCa to immunotherapies. Rather than targeting CXCL12 directly, another report described the entrapment of a small molecule antagonist of its CXCR4 receptor within polyplex nanoparticles [162]. By interrupting tumour–PSC interplay via the CXCL12/CXCR4 axis, these nanoparticles induced multiple immunostimulatory effects, including CD8+ T cell recruitment and a proportional reduction in M2 macrophages, which were potentiated upon co-loading of a microRNA that restored PSC quiescence. This transformation of the tumour microenvironment into an immunogenic milieu holds much potential to sensitise PaCa to IO agents such as checkpoint inhibitors.

Stromal macrophages have also received considerable attention, given their unique functional plasticity that is highly amenable to therapeutic manipulation [163,164]. During pancreatic carcinogenesis, macrophages adopt a pro-tumour M2-like phenotype, leading to adaptations in their secretomes and surface marker profiles that foster immune suppression. However, reports have shown that anti-tumour immunity can be restored through re-education of macrophages, using diverse approaches such as Toll-like receptor (TLR) agonism and kinase inhibition to induce M1-like polarisation [165,166,167,168,169,170]. This strategy has proven successful in various PaCa models, where nano-enabled reprogramming of macrophages has been achieved through direct targeting of these cells or as a downstream consequence of other immunotherapeutic approaches [171,172,173]. Of note, nanoparticles are ideally suited for macrophage targeting given that they are often susceptible to phagocytosis by these cells [174,175]. Exploitation of this vulnerability can therefore allow for the selective delivery of entrapped M1 polarising agents to macrophages. Despite this advantage, potential hurdles exist in that it is now standard practice to formulate therapeutic nanoparticles with a ‘stealth’ PEG corona to evade macrophage recognition and clearance from the circulation. Engineering of nanoparticles with environmentally-responsive properties could provide an innovative solution to this challenge, whereby protective coatings such as PEG are designed to remain intact in the bloodstream followed by detachment upon reaching the tumour site [176,177,178,179]. As demonstrated by Han et al., this can be achieved by grafting PEG chains onto nanoparticles via matrix metalloproteinase-9 (MMP-9)-labile linkers, which are cleaved upon encountering high levels of the protease in the pancreatic tumour microenvironment [180]. Although the purpose of PEG removal in this study was to expose an underlying RGD targeting motif for improved internalisation by tumour cells, the same principles could be applied to unmask M1-polarising nanoparticles in the pancreatic stroma and promote their engulfment by macrophages. Continued efforts to harness the unique capabilities of nanotechnology to re-educate macrophages will form an important future research direction, given that M2-like programming is a poor prognostic indicator in PaCa patients [181,182,183,184]. 

Other cell populations present within the pancreatic tumour stroma include myeloid-derived suppressor cells (MDSCs) and regulatory T cells (Tregs). Despite being of relatively low abundance, these cells can disproportionately contribute to drug resistance via diverse mechanisms that converge in tumour immune escape, such as induction of T cell anergy, M2 skewing of macrophages and suppression of dendritic cell activity [185,186]. Direct targeting of both cell subsets has not been widely pursued by the nanomedicine field, although alterations in their tumour composition have been noted as a secondary effect of other nano-based strategies, leading to favourable outcomes in PaCa models [131,187,188,189,190]. Thus, a clear rationale exists for developing nanoparticles that directly modulate the immunosuppressive functionalities of MDSCs and Tregs, which could effectively prime pancreatic tumours for therapy with IO agents. 

### 3.4. Cancer Stem Cells (CSCs)

CSCs comprise a small pool of pluripotent cells within tumours that are characterised by their unique ability to self-renew and give rise to differentiated progeny. These cells exhibit a remarkably resistant phenotype that can withstand multiple modes of treatment, and their persistence within tumours is believed to sustain malignant growth that eventually results in more aggressive and metastatic relapse. As such, CSCs have been proposed as major drivers of oncogenesis and are considered crucial targets if durable tumour control is to be achieved. Progress in targeting CSCs has been aided by the development of nanomedicines that interfere with CSC biology [191,192,193]. These include a polymeric nanoformulation of the natural xanthonoid α-mangostin, whose application as a free agent is otherwise limited by poor aqueous solubility. Testing of the α-mangostin nanoformulation in the KPC model of PaCa led to reductions in molecular signatures associated with pluripotency and stemness, including CD24, CD133, c-Myc, Oct4 and Nanog protein expression [194]. This was accompanied by a reduction in tumour burden with complete absence of metastatic hepatic nodules. Elsewhere, a variety of other nanoformulations have been shown to alleviate the stem-like characteristics in PaCa models, including copper oxide nanoparticles [195] and superparamagnetic iron oxide nanoparticles loaded with curcumin [196] or a leptin inhibitor [197]. Several formulations have also been functionalised with ligands that engage CSC surface markers such as CD44 and CD133, leading to notable improvements in CSC targeting selectivity [198,199,200,201]. Although these developments are encouraging, nano-assisted strategies for tackling the CSC population are generally lacking throughout the published literature. Intensified research in this area will therefore be of key importance moving forwards for lasting suppression of PaCa.

### 3.5. Altered Physiology

Tumours exhibit a range of physiologic alterations compared to normal tissue, such as a reduction in extracellular pH that renders the microenvironment mildly acidic. This largely results from metabolic adaptations of cancer cells, which show an enhanced propensity to fuel their energy demands through aerobic glycolysis to yield the acidic end product lactate (termed the ‘Warburg effect’). One of the major repercussions of microenvironmental acidification is a reduction in cellular drug influx that can lead to therapy resistance. Weakly basic drugs, which account for a sizeable proportion of all anticancer agents, are particularly affected as they become protonated under acidic conditions to an ionised form that does not readily diffuse across the cell membrane. Clear scope therefore exists for the use of nanoparticle vectors that can shield entrapped cargo from the acidic milieu and provide a route of intracellular entry. In many instances, these have been engineered with ‘smart’ features that take advantage of tumour acidosis to achieve spatiotemporal control over drug delivery. For example, studies by Han et al. showed that surface functionalisation of gemcitabine-loaded nanoparticles with a pH-responsive peptide could markedly improve their binding affinity to tumour cells [202]. This resulted from a conformational change in the peptide upon exposure to low pH, allowing it to form a stable transmembrane helix across the tumour cell bilayer that facilitated nanoparticle uptake. Other studies have also exploited tumour acidity as a release stimulus for nanoencapsulated payloads that could help to overcome resistance due to off-target drug distribution [203,204,205,206,207,208,209,210,211,212]. 

Oxygen deprivation is a further hallmark that commonly distinguishes tumour, from normal, tissue. This characteristic is prominent in PaCa, where hypovascularity together with stroma-mediated compression of existing vasculature create barriers to blood flow that reduce oxygenation. It is well established that hypoxic conditions give rise to many of the major mechanisms of drug resistance, such as anti-apoptotic signalling, immune suppression and induction of EMT [213,214]. Restoration of tumour normoxia has therefore been investigated as a potential therapy, and many studies have shown that nanotechnology can play a vital role in the success of this approach [215,216,217,218,219]. For example, a report by Chen and colleagues described the adsorption of oxygen to fluorocarbon chains on the surface of mesoporous silica nanoparticles, which effectively relieved hypoxia when delivered to PANC-1 pancreatic xenografts [220]. Resistance to sonodynamic therapy could be largely overcome as a result, which relies on sufficient oxygen supply for generation of cytotoxic reactive oxygen species. Similarly, others have shown that hypoxia reversion using calcium peroxide nanoparticles can attenuate resistance to oxygen-dependent photodynamic and sonodynamic therapy in PaCa models [221,222]. These nanoparticles were coated with a pH-responsive polymer that remained intact during circulation but degraded under acidic conditions, ensuring selective exposure of their oxygen-generating core in the locality of the tumour. Taken together, nanotechnology can clearly help to offset resistance either by correcting physiological alterations in tumours, or by exploiting them for on-demand drug release. Aside from pH and oxygen status, interest in other tumour features, such as elevated glutathione levels, has also produced encouraging results that are worthy of further investigation [223,224,225,226,227]. 

### 3.6. Metabolic Factors 

Of the drugs used for PaCa therapy, gemcitabine is particularly vulnerable to metabolic processes that contribute to the emergence of resistance [228,229]. While in systemic circulation, gemcitabine is rapidly inactivated by the enzyme cytidine deaminase, resulting in a half-life of only 15–20 min [230]. To achieve sufficient biological activity, frequent infusions at high dosage are necessary at the expense of increased toxicity. This challenge could be circumvented by coupling gemcitabine to the amphiphilic polymer tocopherol polyethylene glycol succinate (TPGS), to generate a prodrug that spontaneously assembled into micelles under aqueous conditions [231]. Following incubation with recombinant cytidine deaminase, the authors observed a time-dependent degradation of native gemcitabine, with only 11% of the original amount detected after 30 min. By contrast, the deamination of micellar gemcitabine was significantly less pronounced, with 90% remaining at endpoint. Similar findings were also noted elsewhere [180,232,233,234], highlighting the ability of nanotechnology to effectively shield gemcitabine from metabolic inactivation. Interestingly, cytidine deaminase expression can be inhibited by the paclitaxel nanoformulation Abraxane, which may partially account for the improved clinical outcomes upon combining this agent with gemcitabine [235].

A further obstacle associated with gemcitabine therapy is the requirement for intracellular processing of the drug to a pharmacologically active conformation. This involves a series of phosphorylation events mediated by several enzymes whose expression is often dysregulated in PaCa. Hence, multiple opportunities exist for insufficient drug processing, leading to chemoresistance. In particular, monophosphorylation of gemcitabine by deoxycytidine kinase (dCK) is regarded as the critical rate-limiting step towards production of the bioactive triphosphate metabolite [236]. Given that dCK downregulation is a frequent occurrence in PaCa that correlates with gemcitabine insensitivity [237], studies have endeavoured to bypass this step through direct delivery of gemcitabine monophosphate to the tumour site. This gemcitabine derivative has been successfully formulated within a range of nanoparticle platforms that have demonstrated significant antitumour efficacy in PaCa models [151,189,238]. It is also notable that, even in cases where the parent gemcitabine molecule has been entrapped in nanoparticles, both high and prolonged accumulation of the active triphosphate metabolite has been achieved in tumour tissue compared to the free drug [180,232]. These observations are a likely reflection of the improvements in metabolic stability, tumour localisation and cellular uptake of gemcitabine when loaded within nanoparticles. Similarly, resistance often results from inefficient conversion of irinotecan to bioactive SN-38 due to limited expression of the metabolising enzyme carboxylesterase in humans. However, studies have shown that direct delivery of SN-38 within nanoparticles leads to carboxylesterase-independent efficacy in PaCa models, while overcoming solubility and toxicity issues that limit the application of SN-38 in free format [239].

Rewiring of amino acid metabolism in PaCa can also induce resistance to many IO therapies by exerting suppressive effects upon antitumour immunity. Enhanced tryptophan catabolism represents a classic example of metabolic reprogramming in PaCa, which mainly results from overexpression of indoleamine 2,3-dioxygenase-1 (IDO1) that catalyses the breakdown of this essential amino acid to kynurenine. In turn, local tryptophan depletion, combined with accumulating levels of metabolites, leads to a dampening of both innate and adaptive antitumour responses [240,241]. Efforts to remedy this metabolic alteration have yielded a wealth of nanoplatforms encapsulating IDO1-targeted agents, such as small molecule inhibitors or siRNA, often in combination with another immunomodulatory cargo [242,243,244,245]. These have proven highly effective in mobilising antitumour immunity, as demonstrated by the development of a hybrid lipid-mesoporous silica nanoformulation encapsulating the IDO1 inhibitor indoximod and the ICD-inducer oxaliplatin [190]. Treatment of orthotopic KPC allografts with this nanoformulation increased the ratio of CD8+ T cells to Foxp3+ Tregs, leading to significant reductions in tumour volume and metastatic foci. Importantly, these effects translated into a survival benefit versus free drug treatment, which could be explained by data confirming the enhanced circulation half-life and tumour biodistribution of the nanoparticles. 

### 3.7. Suboptimal Receptor Agonism

There is an ever-increasing emphasis on multivalency in the development of new therapies as many targets require receptor clustering to achieve sufficient agonism and aid internalisation. Indeed, receptor clustering has been suggested, as this is a way cells may regulate their response to certain stimuli and potentially develop resistance [246,247]. Nanoparticles offer a convenient platform for attachment of existing monovalent therapies in close enough proximity to induce receptor clustering [248]. This has been observed with various multimeric receptors, including epidermal growth factor receptor (EGFR). Attachment of EGFR targeting ligands to the surface of nanoparticles has been shown to aid the targeting of a given payload [249,250]. Numerous antagonistic antibodies targeting EGFR, such as cetuximab, are on the market but only a small proportion of patients respond to therapy [251]. An anti-EGFR liposome containing doxorubicin is currently undergoing clinical trials, therefore it will soon be ascertained if the multivalent targeting of EGFR aids internalisation of an entrapped payload and translates to clinical benefit [252].

Receptor-targeted nanoparticles can also enable novel immunotherapy strategies. Perica et al. used iron dextran nanoparticles that could activate a T cell receptor (TCR) in T cells via a magnetic field, generating proliferating T cells that recognised a grafted tumour and blocked its development in vivo [253]. In a different approach, Smith et al. described a method of rapidly programming T cells in vivo using TCR-targeting nanoparticles containing a DNA payload coding for a chimeric antigen receptor (CAR). Use of these nanoparticles resulted in complete elimination of the tumours in animal models [254]. Given the immunosuppressed nature of PaCa it is likely that such CAR technologies targeting antigens could offer benefits [255].

**Figure 3:**
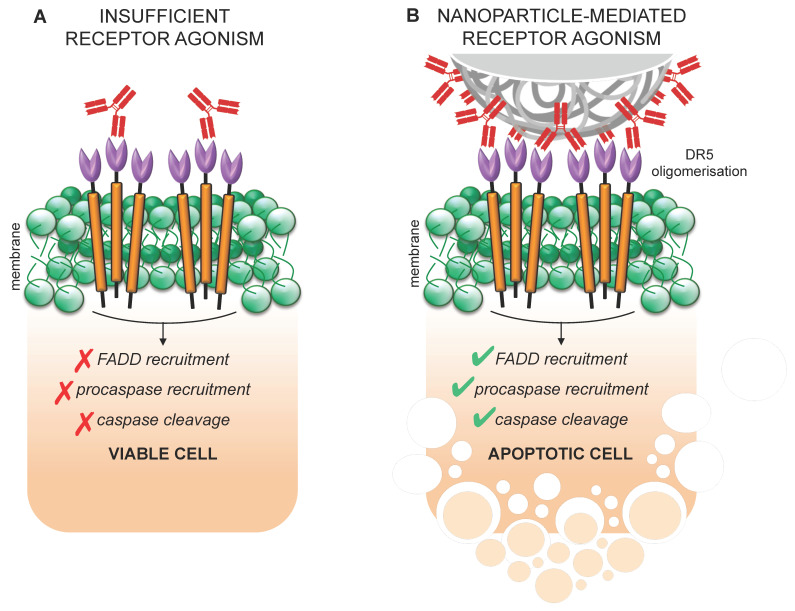
Multivalent antibody presentation on the surface of nanoparticles facilitates receptor activation. Insufficient agonism of target receptors represents a common mode of resistance to many therapies, as observed with antibodies directed against DR5, for example. (**A**) When applied in free format, antibodies show limited ability to induce oligomerisation and cross-linking of DR5 to the extent required for downstream signal transduction. (**B**) However, the concentrated display of antibodies on a nanoparticle surface allows for multivalent engagement of DR5 beyond the threshold needed for receptor activation. Signalling is initiated in response, involving recruitment of FADD and activation of initiator and executioner caspases that mediate apoptotic cell death.

Perhaps the best examples of therapeutics that have taken advantage of multivalency have been antibodies and recombinant proteins targeting death receptor 5 (DR5). The endogenous ligand of DR5, Tumour Necrosis Factor α-related apoptosis-inducing ligand (TRAIL), exists in both membrane bound and soluble forms in nature with the former being much more potent than the latter. Multiple formats of monovalent or bivalent death receptor targeting therapies have gone to clinical trial but failed to produce adequate benefit to warrant use in the clinic [256]. Recent attempts at therapeutically targeting DR5 mostly involve presenting the ligand in a multivalent format to encourage DR5 clustering. Nanoparticles offer a convenient platform to attach mono- or bivalent ligands in close proximity to encourage receptor clustering (Figure 3). Collectively this work demonstrates improved antitumour effects in multiple cancer models, including using TRAIL itself or the anti-DR5 antibody conatumumab [257,258,259,260,261]. In the case of PaCa, it has been demonstrated that targeting DR5 on tumour-associated endothelium can reduce interstitial fluid pressure facilitating increased delivery of nanoformulations [262]. Many other targets may also exhibit an increased signal when targeted with a multivalent ligand where conjugation of a monovalent ligand to a nanoparticle could offer a convenient platform to create multivalency [263,264].

### 3.8. Antiapoptotic Proteins

Chemoresistance can result from the overexpression of antiapoptotic proteins within the DR5 pathway, such as Fas-associated death domain (FADD)-like IL-1b-converting enzyme-inhibitory protein (FLIP) [265]. FLIP is overexpressed in many cancers and is associated with a poor prognosis. Its overexpression is associated with reduced efficacy of chemotherapeutics used in the clinic, such as oxaliplatin and 5-FU [266]. High FLIP expression also causes resistance to apoptosis targeting therapies such as TRAIL receptor agonists [267]. Numerous therapies have been shown to downregulate FLIP, resulting in increased sensitisation to chemotherapies and TRAIL receptor-targeting therapies [265]. Nanoparticles can help to overcome this resistance and have been used to deliver FLIP downregulating agents, such as camptothecin, aiding sensitisation to therapy [259,261,268].

B cell lymphoma 2 (Bcl-2) and myeloid cell leukaemia (Mcl-1) are antiapoptotic members of the Bcl-2 family of proteins that bind to their proapoptotic family members, thereby preventing mitochondrial outer membrane permeabilisation (MOMP), the release of cytochrome c and second mitochondrial activator of caspase (SMAC) [269]. The antiapoptotic members of the Bcl-2 family are attractive targets for cancer therapy. In 2020, the Bcl-2 specific inhibitor venetoclax was approved for use by the FDA for the treatment of chronic lymphocytic leukaemia and small lymphocytic lymphoma [270], whereas earlier attempts at more broad-spectrum inhibition of antiapoptotic Bcl-2 family members showed much higher levels of toxicity [271]. Nanotechnology can offer benefit through limiting systemic toxicity; Tannan et al. demonstrated that the use of the Mcl-1 specific inhibitor S63845 and venetoclax as free drug co-therapies caused haematological toxicities and weight loss in mouse models. They successfully mitigated these toxicities through entrapment of these agents in nanoparticles while also allowing for a 3.5- to 6.5-fold reduction in overall drug dose [272]. While the full potential of nanotechnology in this field has not been realised, clearly this approach offers a useful strategy to reduce systemic toxicities, which should increase the demographic of patients able to receive the entrapped payloads, ultimately resulting in better survival outcomes.

## 4. Conclusions and Future Perspectives

As one of the most difficult-to-treat malignancies with a survival rate that has scarcely improved over several decades, the identification of new therapeutic strategies for PaCa remains a leading priority. The application of nanomedicine represents a significant step towards realising this goal, which has proven capability to overcome mechanisms of resistance that ultimately lead to therapy failure and relapse in PaCa patients. Efforts in this area to date have resulted in two clinically approved nanomedicines for PaCa therapy, with numerous others demonstrating promise at various stages of development. One commonality across many of these platforms is their containment of a sole chemotherapeutic payload, such as paclitaxel and irinotecan in the case of Abraxane and Onivyde, respectively, which will be subject to much adaptation moving forwards. Initiatives to co-load multiple cargoes within a single nanoparticle platform are expected to gather pace, to overcome issues with current combination regimens in the clinic and also allow for the simultaneous and aggressive targeting of multiple aspects of PaCa pathophysiology. This will be greatly facilitated by the breadth of core materials now available for nanoparticle construction, and hybrid compositions may soon take precedence to enable packaging of drugs with disparate structural and physicochemical properties. However, although the formulation of multidrug nanoparticles offers clear benefits, these are partially offset by the inherent complexity of the final product and the need for extensive optimisation to achieve precise ratiometric control over drug loading. A key consideration will be to ensure that manufacturing practices for these nanoparticles provide a sufficient degree of homogeneity and reproducibility, to satisfy regulatory requirements and expedite their clinical translation. Another important objective of future work will involve payload diversification. While conventional chemotherapies will continue to hold a fundamental role in treatment, other payloads will be increasingly explored in line with our evolving understanding of PaCa biology and the processes that contribute to resistance. Leading candidates in this respect will include agents with immunostimulatory properties, driven by the unprecedented success of IO approaches such as checkpoint inhibition that have revolutionised cancer care in recent years. Moreover, attention must also be centred on the development of appropriate preclinical models in which to test these nanoformulations. Recent modelling advances include those based on 3D organoids and humanised mice that can recapitulate many features of PaCa, and continued innovation in this area will assist the translation of novel nanoformulations to the clinic. Collectively, these efforts will establish a rich pipeline of therapeutic nanomedicines that, with time, could lead to a radical transformation in the outlook for PaCa patients.
